# A dung beetle that path integrates without the use of landmarks

**DOI:** 10.1007/s10071-020-01426-8

**Published:** 2020-09-09

**Authors:** Marie Dacke, Basil el Jundi, Yakir Gagnon, Ayse Yilmaz, Marcus Byrne, Emily Baird

**Affiliations:** 1 Lund Vision Group, Department of Biology, Lund University, 22362 Lund, Sweden; 2 School of Animal, Plant and Environmental Sciences, University of the Witwatersrand, Johannesburg, South Africa; 3 Biocenter, University of Wuerzburg, 97074 Wuerzburg, Germany; 4 Department of Zoology, Stockholm University, 10691 Stockholm, Sweden

**Keywords:** Navigation, Homing, Path integration, Search, Dung beetle, *Scarabaeus*

## Abstract

Unusual amongst dung beetles, *Scarabaeus galenus* digs a burrow that it provisions by making repeated trips to a nearby dung pile. Even more remarkable is that these beetles return home moving backwards, with a pellet of dung between their hind legs. Here, we explore the strategy that *S. galenus* uses to find its way home. We find that, like many other insects, they use path integration to calculate the direction and distance to their home. If they fail to locate their burrow, the beetles initiate a distinct looping search behaviour that starts with a characteristic sharp turn, we have called a ‘turning point’. When homing beetles are passively displaced or transferred to an unfamiliar environment, they initiate a search at a point very close to the location of their fictive burrow—that is, a spot at the same relative distance and direction from the pick-up point as the original burrow. Unlike other insects, *S. galenus* do not appear to supplement estimates of the burrow location with landmark information. Thus, *S. galenus* represents a rare case of a consistently backward-homing animal that does not use landmarks to augment its path integration strategy.

## Introduction

**Fig. 1:**
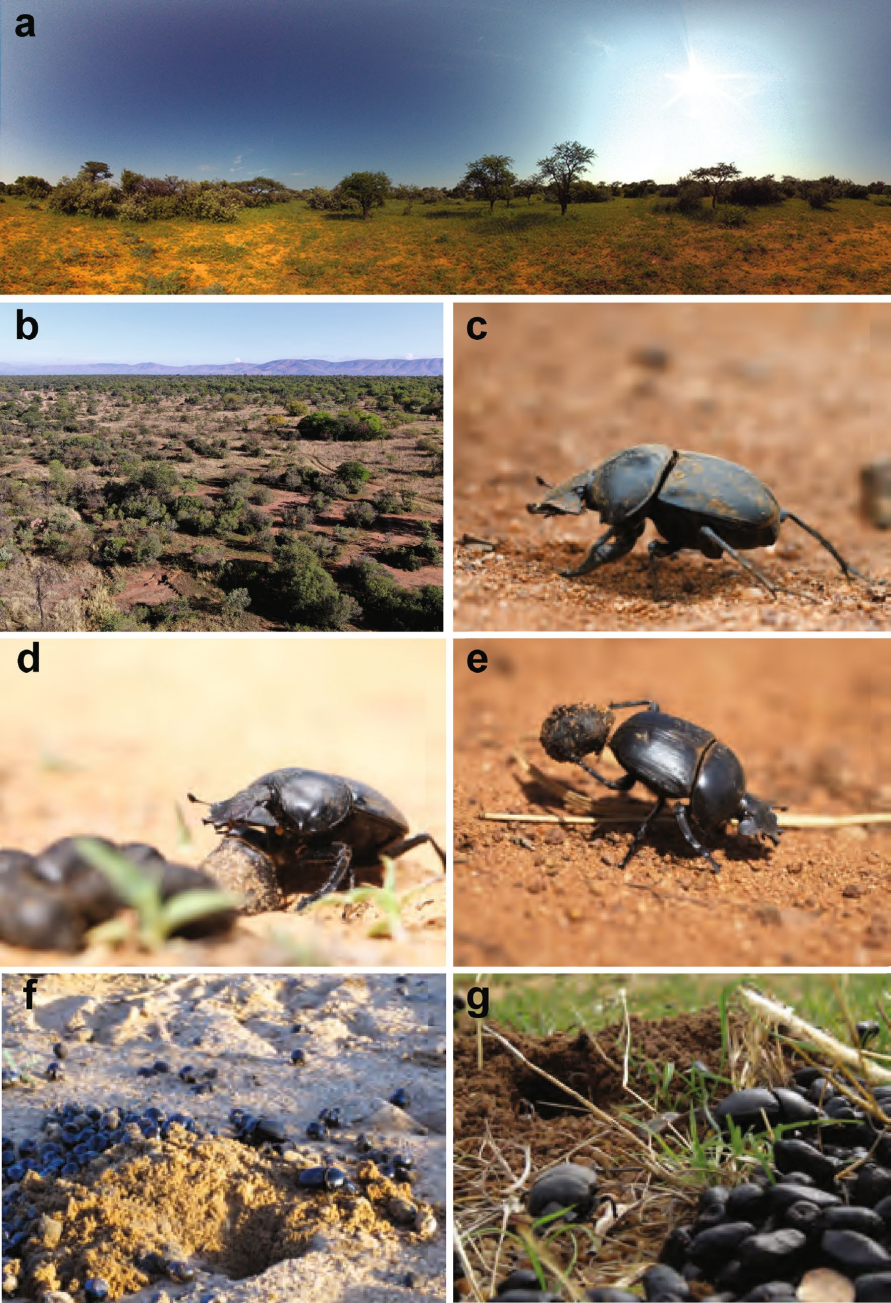
The habitat and foraging behaviour of *Scarabaeus galenus*. Panoramic (**a**) and overhead (**b**) images of the natural woodland-savannah habitat of *S. galenus*. The beetle *S. galenus* walking towards a food source (**c**), collecting an antelope dung pellet (**d**) and carrying it back to its burrow (**e**). *S. galenus* build their burrows in open sandy areas (**f**) or in more cluttered grassy areas (**g**), often with a large pile of antelope pellets in a midden nearby

Once attracted to fresh dung, most dung beetle species stay at the pile, burying themselves in or under it. A smaller proportion of beetles form the dung into a ball and roll it away (Cambefort and Hanski 1991), while even fewer species take a different strategy; upon finding a dung pile, they first leave and start to dig a burrow some distance away (Halffter and Matthews 1966; Monteith and Storey 1981; Scholtz 1989). Once complete, the beetles provision their burrow by making repeated trips to the pile—which may be a dung pat, a collection of antelope pellets or detritus—transporting a piece back home each time. One of these provisioning species is *Scarabaeus galenus* (Fig. 1), which has anecdotally been observed shuttling back and forth over 30 times between a dung source and its burrow in a single morning (Ybarrondo and Heinrich 1996; MD and EB personal observation). In addition to being a rare example of a homing dung beetle, *S. galenus* is remarkable because, unlike all homing animals described to date, it consistently walks backwards when transporting food to its burrow (Fig. 1e). This highly unusual behaviour means that these beetles both leave and approach their burrow while facing away from it.

Locating a tiny (~ 2 cm diameter) and visually inconspicuous burrow entrance from the ground-level perspective of a foraging beetle is no simple task, particularly considering that *S. galenus* achieve this facing backwards. *S. galenus* must therefore have a robust and precise strategy to repeatedly find their way back home. Foraging ants face a similar challenge (albeit mostly facing forwards), with their navigational capacity being the focus of intense research for over a century. Ant species that inhabit relatively featureless environments, such as the salt pans of northern Africa, find their way home by computing a straight-line trajectory that ends at the position of their nest (Wehner 2003). They do this using a strategy called path integration, which calculates the homeward trajectory from the distance (using step counting and/or optic flow) and directional (using celestial compass information) information acquired during their outbound trip (Pfeffer and Wittlinger 2016a; Ronacher and Wehner 1995; Wehner and Müller 2006; Wittlinger et al. 2006, 2007). A common test for path integration is to displace the animal to an unfamiliar location while it is attempting to return home. If the displaced animal travels in the direction and for the distance where home would have been and fails to account for the passive displacement, one can safely conclude that it uses path integration (Heinze et al. 2018).

Ant species that inhabit more cluttered environments, like the pine forests of Greece or the vegetated deserts and bushlands of Australia, in addition to path integration, use landmarks or the skyline panorama to find their way back home (Cheng et al. 2009; Fleischmann et al. 2018; Graham and Cheng 2009; Zeil et al. 2014). Naturally, the relationship between landmarks and the nest needs to be learned in order to be useful for homing. To facilitate this, naive foragers perform well-structured learning walks during which they repeatedly face the nest from a wide range of vantage points (Collett and Zeil 2018; Freas et al. 2019). Such nestward views are obtained by walking in spirals around the nest, by walking in small circles (volts) along the outward path, or by performing pirouettes around the vertical body axis during the learning walk (Fleischmann et al. 2016, 2017, 2018; Müller and Wehner 2010; Wystrach et al. 2014). After performing one or more learning walks, the ants set out to forage. Even during this outbound path, many species frequently turn to look back at the nest as they move away from it (Jayatilaka et al. 2018; Müller and Wehner 2010; Wystrach et al. 2014), probably to also acquire terrestrial cues along the foraging route (Nicholson et al. 1999; Zeil 2012). Over time, the paths of the first few foraging journeys are quite tortuous but become straighter as the number of outbound trips increases (Fleischmann et al. 2016; Müller and Wehner 2010; Wehner et al. 2004).

Navigators active in landmark-rich environments tend to follow their path integrator only for some distance when returning home, before prioritising landmark-based orientation for the remainder of their journey (Schultheiss et al. 2016). Interestingly, ants are still capable of returning to their nest whilst walking backwards, which is sometimes necessary when transporting particularly heavy food items. In this case, they will, from time to time, drop their bulky forage to walk in circles, or in some other way turn to face in the direction of their nest (Ardin et al. 2016; Pfeffer and Wittlinger 2016b; Schwarz et al. 2017), indicating that they may be using landmark-based information to find home. Once close to the nest, ants can also use olfactory cues to locate its entrance (Steck 2012; Steck et al. 2009, 2011). If, however, they fail to locate their nest at the end of their foraging journey, ants typically initiate a systematic search characterised by increasing loops around the theoretical position of their fictive nest (Schultheiss and Cheng 2011; Müller and Wehner 1994; Wehner and Srinivasan 1981).

Although the navigational abilities of a broad range of species of ants living in different types of environments have been intensely and fruitfully studied, our understanding of the homing strategies of other terrestrial insects, with different styles of locomotion and natural history, remains limited. Here, we describe the navigation behaviour of the backward-homing dung beetle, *S. galenus* and explore the strategy it uses to locate its burrow. We find that, if homing beetles are moved a small distance within their normal foraging area, they travel in a direction where the burrow should be, had they not been displaced, seemingly ignoring landmark-based information. If they are moved to a completely unfamiliar location while attempting to return home, these beetles also continue on their original bearing as if no displacement had occurred. Upon reaching the expected position of the fictive burrow, they initiate a search around this point in space. These results suggest that the dung beetle *S. galenus* relies exclusively on path integration to locate the position of its burrow. We discuss these findings in the context of the natural ecology and evolutionary history of these animals.

## Materials and methods

### Animals and experimental site

All experiments were carried out with the diurnal dung beetle *Scarabaeus galenus* in its natural habitat at Thornwood Lodge in Bela Bela, Limpopo, South Africa (27.95° E, 24.78° S; Fig. 1a, b). Experiments were performed in the morning, in February and November from 2014 to 2017. *S. galenus* were collected by hand or by using pit-fall traps at the *Adventures with Elephants* reserve in Bela Bela. Before and between experiments, the beetles were kept in plastic bins filled with soil (30 cm × 22 cm × 22 cm) and fed with fresh cow dung. To avoid pseudo-replication, individual beetles were marked with a number on their thorax using correction fluid.

### Foraging paths in S. galenus

To investigate the foraging behaviour of *S. galenus* (Fig. 1c), a small number (5–10, typically) of individuals were released on a flat, sandy, experimental area early in the morning (~ 06:30–09:30 a.m.) and allowed to search for and collect fresh antelope dung (small round pellets ~ 1 cm in diameter, Fig. 1d, e) placed on randomly dispersed petri dishes. The piles of fresh pellets stimulated the beetles to dig burrows within the experimental area. Once a beetle started to dig a burrow (Fig. 1f, g), the feeder was moved to a distance of 125 cm away (measured from the center of the burrow entrance to the edge of the petri dish), to ensure that all beetles foraged over the same distance in all experiments. The first 10 foraging paths made by a beetle between its burrow and the feeder were filmed from above using a Sony HDR-HC5E Handycam fitted with a 0.42 × wide angle lens and attached to a tripod (this setup was also used to film all paths in the following experimental trials). In another set of experiments, as the beetle headed to the feeder for the 7th time, its burrow was covered with sand and the area swept with a brush to remove potential olfactory cues. The path taken by the returning beetle once it left the feeder, as well as the first 4 min of its search around the closed burrow, was filmed from above.

### Displacement experiments

Upon reaching the feeder for the 7th time, the petri dish (with the beetle on it) was displaced 50 cm in one of four directions: laterally (that is, to the right or left of the feeder, perpendicular to the burrow–feeder axis) or translationally (that is, towards or away from the burrow). In this set of experiments, as well as in all experiments described below, each beetle was allowed to make at least 6 foraging runs before they were manipulated to ensure that they were accurately navigating between their burrow and the feeder. Before releasing the displaced beetles, their burrows were covered over with sand and the ground carefully brushed to remove potential olfactory cues. The path taken by the beetle when searching for its burrow after the displacement was filmed for 4 min (the same cut off was used also in the following experimental trials). In an additional test, some beetles were also displaced 50 cm towards their open burrow.

### Zero-vector state beetles

These beetles were picked up just before entering their burrows after their 7th visit to the feeder—by carefully placing a white opaque plastic container on top of the beetle as it walked over a sandpaper covered plate placed just outside the burrow. The beetles (under the container, on the sandpaper) were then displaced back towards the former position of the feeder (that had been removed as soon as the beetle had walked more than 20 cm away from it). Here, the plate was carefully placed on the ground and the plastic container removed to release the beetle back where the feeder had been located. Before the beetles were released, their burrows were covered over with sand and the area brushed to remove potential olfactory cues.

### Transfer experiments

Beetles were picked up during their 7th visit to the feeder (by carefully placing a white opaque plastic container on top of the feeder) and transferred about 30 m away from the original burrow to a different experimental arena surrounded by a different array of natural landmarks unfamiliar to the beetles. The transfer took approximately 30 s, after which the feeder was carefully placed on the ground and the plastic container removed to release the beetle. The beetle’s subsequent path and search for its burrow was filmed for 4 min. To control for possible effects of the transfer on the beetle’s navigational performance and to generate a performance baseline for this experimental condition, foraging beetles arriving at a feeder at the original experimental site were also covered with the plastic container and transported for 30 m (15 m away and 15 m back) for a duration of ~ 30 s before being placed back on the ground at the exact same position as from where they had been picked up. Before releasing these beetles, their burrows were covered over with sand and the ground carefully brushed to remove potential olfactory cues.

### Analysis

All analysis and data management were done using Julia (Julia: A Fresh Approach to Numerical Computing. Jeff Bezanson, Alan Edelman, Stefan Karpinski, Viral B. Shah. (2017) SIAM Review, 59: 65–98. https://doi.org/10.1137/141000671), unless stated otherwise.

The paths taken by beetles in each of the experiments were tracked using a custom-made Matlab (Mathworks Inc.) software (facilitating but not alleviating the task of manually detecting the location of the beetle in the frame). Pixel coordinates representing the beetle’s position were converted to real world coordinates using the Camera Calibration toolbox in Matlab, which also accounted for any distortion in the images caused by the camera optics. The coordinates of the beetle’s trajectory were smoothed using a parametric spline from the Dierickx Fortran library) with a smoothing factor of 500 and an order of 2. The smoothing spline removed some of the erratic movements of the beetle as well as compensated for any human errors accumulated during the tracking phase. Path straightness was calculated as the distance between the ends of the path divided by the length of the path.

The turning point (for three examples, see Fig. 4b) was defined as the point where the direction of the trajectory deviated from its main direction by more than 60°. The process was divided into two steps. Step (1) Detecting the segment containing the turning point: The derivative of the spline was calculated at the spline’s knots (where the sequential smoothing polynomials connected). The derivative was used to calculate the direction of the beetle. When the direction deviated by more than 60° between two sequential knots, the segment between those two knots was defined as containing the turning point. Step (2) Detecting the turning point: The turning point was defined as the first point along the identified segment at which the beetles heading (calculated from the derivative) deviated from the direction at the first knot by more than 60°.

The center of search was calculated as the mean coordinate of the track excluding the homing part—that is, from the turning point until the end of the track or until 4 min have passed from the beginning of the search.

The fictive burrow’s position was calculated as the location where the burrow would have been if it had been displaced with the beetle. In the displacement experiments, the location of the feeder before and after its displacement was recorded. The direction and distance by which the feeder was displaced was applied to the location of the real burrow, resulting in the position of the fictive burrow. In the transfer experiments, the real burrow was completely outside the viewing frame of the camera where the beetle was released. We measured the azimuth of the real burrow relative to the feeder and the distance between the feeder and this burrow. In the transfer location, we included a visual marker of magnetic north and used that to determine the theoretical location of the fictive nest.

### Statistical analyses

Linear Models (LM) were used to evaluate the effect of displacement on the resulting turning points and centers of search. Generalized Linear Mixed Models (GLMM) were used when the response was non-linear and the same individual beetles were repeatedly tested (e.g., the effect of repeated runs on the straightness and speed of the trajectories). In addition, we used a permutation test for comparing variance between nominal groups (displaced versus not displaced beetles) and a simple one sample *t* test to compare the fictive nest to the turning points or centers of search.

In all comparisons, we chose to test the two spatial dimensions (the *x* and *y* axis) separately. Apart from the added benefit of simpler statistical methodology, we base this decision on the fact that all the displacements were along only one of these two dimensions (i.e., no beetles were displaced diagonally). Since there is no reason to believe that there is an interaction between turning points and centers of search, we kept the analysis of those two indicators separated as well.

Unless otherwise stated, reported values are mean ± SD. The full width at half maximum (FWHM) in Figs. 4, 5, 6, 7 represents the width of a fitted 2D Gaussian distribution at half of its maximum amplitude.

## Results

### General description of foraging behaviour

*Scarabaeus galenus* is frequently found foraging in antelope dung middens or on sparsely distributed piles of pellets in savanna and woodland-savanna areas across South Africa (Fig. 1a, b). Within these landmark-rich environments, *S. galenus* make their burrows (holes dug into the soil) wherever the substrate seems soft enough for them to dig into, including open sandy areas or grassy plains (Fig. 1f, g), as well as under stones or at the base of plants. After locating a fresh pile of dung, the beetles will first start to dig their burrow a short distance away (150 cm [83 cm 245 cm] (median [interquartile range], *n* = 38)). After some time (typically 5 min–20 min, MD and EB personal observation), they will then return to the pile, pick-up individual pellets in their hind legs and carry them backwards towards their burrow (Fig. 1e), where they drop or push the pellets down the entrance. *S. galenus* will repeatedly shuttle back and forth between a pile of antelope pellets and its burrow until the food source is depleted or until it ceases to forage (presumably when enough dung has been gathered). Excavated burrows have been found to contain up to 50 pellets and it is common to see individual beetles performing more than 10 foraging trips, with up to 125 trips having been observed (MD and EB personal observation). On its way to the food source, *S. galenus* walks forward on six legs (Fig. 1c and then reverses back to the burrow on four legs with the pellet held tightly between the hind legs (Fig. 1e).

**Fig. 2:**
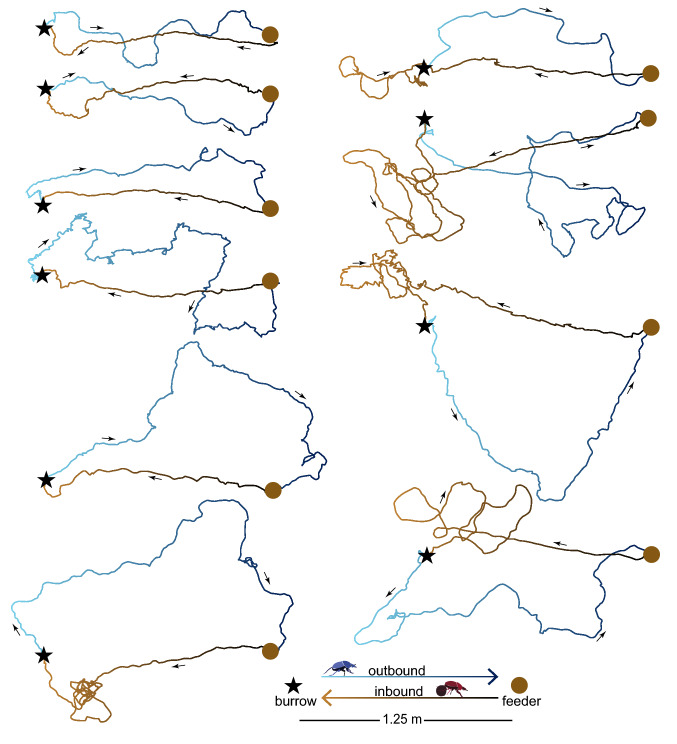
The first foraging trip of individual *Scarabaeus galenus*. Tracks of 10 individual beetles, each making their first foraging trip to the feeder (brown circle), placed 1.25 m from their burrow (black star). Outbound tracks—where the beetle is walking forwards on 6 legs from the burrow to the feeder—are indicated with blue lines. Inbound tracks—where the beetle is walking backwards carrying a pellet from the feeder to the burrow—are indicated with brown lines

**Fig. 3:**
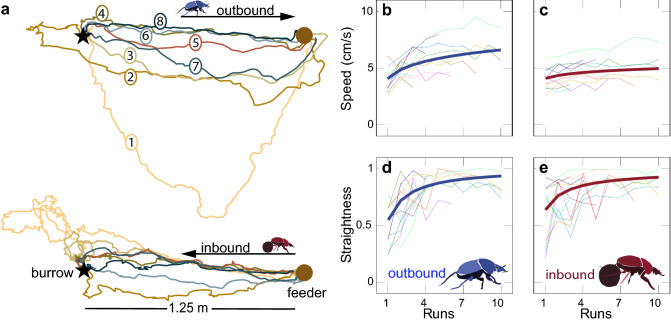
Repeated foraging paths of *Scarabaeus galenus*. **a**) Eight consecutive paths (numbered in order) made by one individual beetle walking forwards outbound from the burrow (black star) to the feeder (brown circle) and the corresponding (same color) inbound paths walking backwards with a pellet (the inbound and outbound paths were separated for clarity). The average speed (**b**, **c**) and straightness (**d**, **e**) of repeated outbound (**b**, **d**) and inbound (c, e) runs of 11 individual beetles foraging between the feeder and the burrow. The thick lines are the predicted speed and straightness calculated from the fitted GLMM

Once the burrow is complete, the first outbound foraging trip is often quite tortuous but nonetheless directed in the approximate direction of the previously discovered food source (Fig. 2). This is not surprising given that the beetles dig the burrow *after* they have located a suitable food source and indicates that, even while digging, they maintain some memory of the direction of the food source. As the beetles make repeated trips to and from the food source, their paths become straighter and more directed (GLMM with a beta family and logit link function *P* < 0.001) and their walking speed increases (GLMM with a beta family and logit link function *P* < 0.001, Fig. 3). However, the speed of the outbound trips, where the beetles move forwards on six legs, increases more than the speed of homebound trips, when the beetles are walking backwards on four legs while carrying a pellet of dung between their back legs (*P* < 0.001).

**Fig. 4:**
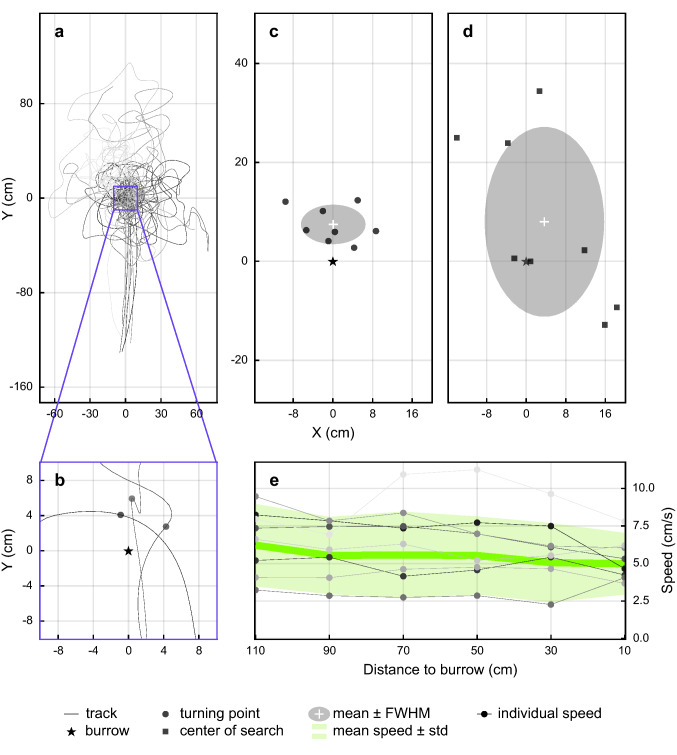
The turning point and systematic search of *Scarabaeus galenus*. **a** Tracks of 7 individual beetles returning from the feeder (*X* = − 125 cm, *Y* = 0 cm) to their burrow (*X* = 0 cm, *Y* = 0 cm) that had been covered by the experimenter (the varying shades of the tracks indicate paths of different individuals). The tracks, that are initially straight and clustered about the *X* axis, transition into a series of loops after the beetles perform a distinct side-ways deflection or ‘turning point’, illustrated for 3 tracks in (**b**). The position of the turning points (dark grey circles, **c**) and the center of search (dark grey squares, **d**) relative to the covered burrow entrance (*X* = 0 cm, *Y* = 0 cm) defined for the search paths of 8 individual beetles. Light grey shaded areas indicate mean ± full width at half maximum (FWHM). **e** The speed over distance of individual beetles (binned and averaged at 10 cm intervals) as they approach the burrow. The mean and standard deviation of each binned point are marked with a solid green line and shaded area, respectively

If the burrow entrance was covered by the experimenter (or if the beetle missed the entrance on its return, which occasionally happens even in natural foraging situations), these beetles initiated a systematic search pattern (Fig. 4a, b). This search started with an obvious side-ways deflection from an otherwise straight homing path—defined here as the ‘turning point’—(Fig. 4b), followed by a number of loops (Fig. 4a). The mean turning point was located 0.1 ± 6 cm to the right of the closed burrow entrance and 7 ± 4 cm beyond it (*n* = 8) (Fig. 4c). The mean center of search (i.e., the most frequently visited point in space of the extended search) coincided well with the burrow location, 4 ± 11 cm to the right of the closed burrow entrance and 8 ± 17 cm beyond it (*n* = 8) (Fig. 4d), indicating where the beetles expected to find their home. The beetles transported the pellet from the feeder to the nest at a speed of 5.7 ± 0.7 cm/s (*n* = 8), with a minor deceleration over the last 50 cm before the burrow (Fig. 4e).

### Path-integrating beetles do not compensate for passive displacements.

**Fig. 5:**
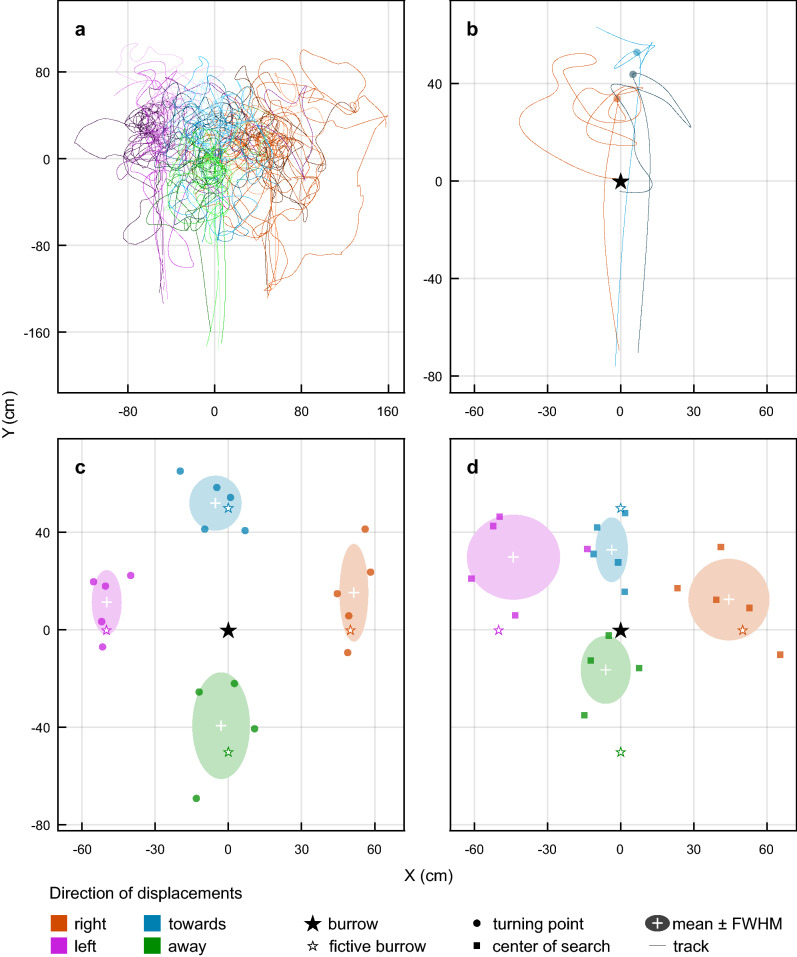
The effect of passive displacement on the search path in *Scarabaeus galenus*. **a** The paths and subsequent searches of beetles returning home from a feeder (originally positioned at *X* = 0 cm, *Y* = − 125 cm) that had been displaced 50 cm to the right (*X* = 50 cm, *Y* = − 125 cm; red traces), left (*X* = − 50 cm, *Y* = − 125 cm; purple traces), away (*X* = 0 cm, *Y* = − 175 cm; green traces) or towards (*X* = 0 cm, *Y* = 75 cm; blue traces) of the burrow position (*X* = 0 cm, *Y* = 0 cm). **b** Detail of the change from straight line run to search and the turning point (colored circles) for the runs of three beetles after being displaced 50 cm towards the open burrow (black star). The turning points (circles, **d**) and centers of gravity (squares, **e**) of the search paths of the displaced beetles in the conditions described in (**a**) with respect to the location of the real burrow (black star). Colored stars indicate the location of the fictive burrow for each condition—that is, the theoretical location of the burrow according to the beetles’ path integration vector—that is, the point at the same relative distance and direction from the feeder after displacement as the original burrow had been before displacement. White crosses and shaded areas indicate the mean and full width at half maximum (FWHM) for the turning points (**c**) or centers of search (**d**) for each of the displacements

When collecting a 7th pellet, the beetle and feeder were passively displaced 50 cm either laterally (to the left or right of the feeder) or translationally (towards or away from the burrow). From this new location, the beetles immediately set out with a dung pellet from the release location in a straight line parallel to the original homewards path before initiating a search (Fig. 5a). For all conditions, this significantly shifted the position of the turning points as well as the center of the search (Fig. 5b, c; Table 1). We tested the effect of the displacement on the location of both turning points and centers of search (for each axis separately) with a linear model. The resulting location of the turning points as well as the centers of search linearly depended on the displacement. This relationship was almost one-to-one (turning points: (*x* = 0.93, *y* = 0.89), centers of search: (*x* = 0.94, *y* = 0.87)) and highly significant (*P* < 0.001 for both points and axes, *n* = 27).

**Table 1:** The effect of passive displacements of beetles at the feeder on their subsequent search location

Displacement at the feeder	*x*, *y* position of turning point (cm, mean ± SD) Burrow at 0,0	*P*_fictive burrow_ *** = *p* < 0.001 ns = *p* > 0.05	*x*, *y* position of center of search (cm, mean ± SD) Burrow at 0,0	*P*_fictive burrow_ ns = *p* > 0.05	*n*
None	0 ± 6, 7 ± 4	ns, ***	4 ± 11, 8 ± 17	ns, ns	8
50 cm left	− 50 ± 6, 11 ± 13	ns, ns	− 44 ± 18, 30 ± 17	ns, 0.016	5
50 cm right	51 ± 6, 15 ± 19	ns, ns	44 ± 16, 12 ± 16	ns, ns	5
50 cm away from the burrow	− 5 ± 10, 52 ± 11	ns, ns	− 4 ± 6, 33 ± 13	ns, 0.038	5
50 cm towards the burrow	− 3 ± 12, − 39 ± 21	ns, ns	− 6 ± 10, − 16 ± 14	ns, 0.016	4

The location of the turning points and centers of searches were, for all conditions, centered around the location of their respective fictive burrow, i.e., the site where the burrow should be if it had been displaced along with the beetle (Fig. 5b, c; Table 1). This strongly suggests that *S. galenus* only followed a home vector, obtained by path integration, as if they had not experienced a displacement.

To test how the precision of the home vector (i.e., the spread of the turning points and centers of search) was affected by passive displacements, we centered all displaced groups to their means and ran an one-tailed permutation test (with one million randomly sampled permutations from 10^28^ unique permutations) on the variance of the *x* and *y* values (with *x* values aligned along the feeder–nest axis and *y* values aligned perpendicular to this) of the turning points and centers of search separately (*n* = 27). Although the variance of the turning points of the displaced groups of beetles (*σ*^2^ = 218) was significantly larger than for the unmanipulated (‘none’) control group of beetles along the *y* axis (*P* < 0.001), we saw no difference along the *x* axis, (none *σ*^2^ = 35, displacement *σ*^2^ = 60, *P* > 0.1). This suggests that the distance measured (spread along the *y* axis) by the beetles was more affected by the passive displacement than their perception of direction (spread along the *x* axis). The same analysis showed no significant difference for the centers of search (none *σ*^2^: (*x* = 120, *y* = 303), displacement *σ*^2^: (*x* = 154, *y* = 183), *P* = (*x* = 0.43, *y* = 0.89)), which supports the suggestion that *S. galenus* perceives distance less precisely than direction.

To further test the strength of the home vector, we ran an additional test where 6 beetles were displaced 50 cm towards an open burrow. Three of these beetles fell into their burrow as they ran over it but the other three ran straight past it, even when they were within just a few cm of the open entrance (Fig. 5b). Taken together, these results suggest that the *S. galenus* do not rely heavily on the features of the surrounding environment—such as the skyline panorama or landmarks, or even odor—to locate the position of the burrow, but rather rely on the direction and length of a homing vector.

### Beetles in a zero-vector state do not find their way back home

**Fig. 6:**
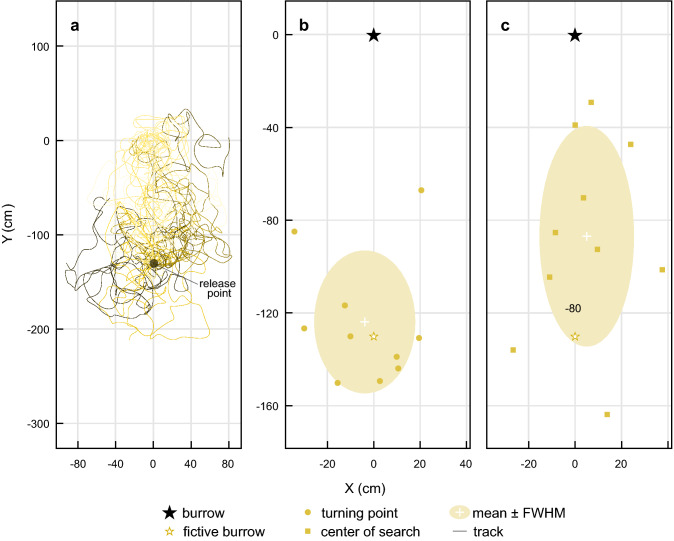
The paths of *Scarabaeus galenus* collected at their burrow at the end of a foraging trip and then moved back to the feeder (zero-vector state). **a** The paths of 10 zero-vector state beetles taken from their burrow (*X*  =  0 cm, *Y*  =  0 cm) and released at the site of the feeder (*X*  =  0 cm, *Y*  =  − 125 cm). The turning point (circles, **b**) and center of search (squares, **c**) of the zero-vector beetles. White crosses and the shaded area indicate the mean and the full width at half maximum (FWHM) of the points. The colored star indicates the site of the fictive burrow—in this case, the theoretical location of the beetles’ burrow according to their path integration vector

In this experiment, homing beetles carrying a dung pellet were collected and covered by an opaque container at the burrow entrance and displaced 125 cm back to the former position of the feeder. In this condition, they would have run off their home vector (i.e., they would be in a ‘zero-vector’ state) and could thus no longer rely on path integration to locate their burrow. Once the container was removed, the beetles immediately set out with their pellet from the release location, but almost immediately initiated a search (Fig. 6a). Again, the turning points, as well as the centers of search, were located around the position of the fictive burrow, that was now the release location of the beetle (turning point − 4 ± 20, − 124 ± 28; center of search 5 ± 18, − 87 ± 43; *x*, *y* cm (fictive burrow at − 125, 0); *n* = 10) (Fig. 6b, c). Although some of the centers of search did occur closer to the burrow, none of the zero-vector beetles ran over the position of their real burrow during the 4 min of recorded search time (Fig. 6a). This further strengthens the observation that the skyline panorama or landmarks do not strongly influence the homing strategy of the beetles.

### Beetles transferred to an unfamiliar environment search for their burrows at the end of their home vectors

**Fig. 7:**
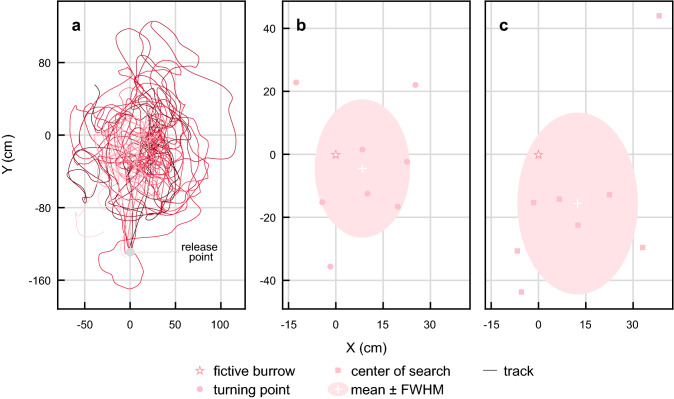
The paths of *Scarabaeus galenus* when displaced to an unfamiliar environment. **a** The paths taken by 10 beetles after being captured at the feeder and released in an unfamiliar environment. The theoretical path integration vector would point to the fictive burrow location at *X* = 0 cm, *Y* = 0 cm. The turning points (circles, **b**) and centers of search (squares, **c**) of the search initiated after the initial straight-line run. White crosses and shaded colored areas indicate the mean and the full width at half maximum (FWHM) of the data. The star indicates the site of the fictive burrow—in this case, the theoretical location of the beetles’ burrow according to their path integration vector

In the next set of experiments, 10 beetles were covered by an opaque container while at the feeder and then transported along with the feeder for ~ 30 s to a novel experimental site ~ 30 m away. Here, the feeder and the beetle were placed gently on the ground and the container removed. As soon as they were released, the beetles started to move backwards along a straight path, carrying a pellet in their hind legs, in a direction that corresponded to the direction of the fictive burrow (i.e., the location of the burrow if it had been moved with the beetle). On covering the appropriate distance approximately, the beetle then initiated a search. (Fig. 7a). The turning point was located 8 cm ± 15 cm to the right of the position of the fictive burrow and 6 cm ± 19 cm before it (*n* = 10). The center of the search was located 12 cm ± 21 cm to the right of the burrow and 13 cm ± 24 cm in front of it (*n* = 10). (Fig. 7b, c). Both these positions in space coincide with the position of the fictive burrow (turning point: *P*(*x* = 0.13, *y* = 0.54); center of search: *P*(*x* = 0.08, *y* = 0.14), one sample *t* test, *n* = 8). Again, these experiments indicate that *S. galenus* rely on path integration, rather than familiar landmarks, to return to their burrow.

As a control for any disturbances caused by covering, transporting and releasing the beetles, another group of beetles were picked up at the feeder and transported ~ 30 m (15 m away, 15 m return) for 30 s before being released at the feeder’s original location. Upon release, these ‘control beetles’ carried their pellet back to the location of their now covered burrow, where they initiated a search. The turning point was located 2 ± 7 cm to the left of the burrow and 6 ± 14 cm after it (*n* = 9). The center of the search was located 1 ± 11 cm to the left of the burrow and 11 ± 11 cm after it (*n* = 9). This confirms that *S. galenus* can still successfully locate its burrow with high precision after having been caught and carried in a closed container.

To further test how the familiarity of the area affects the variance in the beetles’ ability to locate their burrow, we ran a one-tailed permutation test on the variance of the x and y values of the turning points and centers of search for the beetles transported to a ‘novel’ experimental site and for the beetles transported ‘back’ to the original position of the feeder. This did not reveal any significant difference in performance (turning point: *σ*^2^_back_(90 cm^2^, 226 cm^2^), *σ*^2^_novel_(216 cm^2^, 352 cm^2^), *P*(0.09, 0.26); center of search; *σ*^2^_back_(133^2^ cm, 123^2^ cm), *σ*^2^_novel_(375^2^ cm, 655^2^ cm), *P*(0.07, 0.05) (*x*, *y*; *n* = 18)). This further strengthens our observation that *S. galenus* do not use familiar topographical features of the surrounding environment to locate the position of their burrow, but rather use the direction and length of their home vector.

## Discussion

*Scarabaeus galenus* is an unusual example of a navigating dung beetle and is currently the only described example of a navigator that systematically moves backwards when homing. The beetles’ first outward trajectory can be quite long and tortuous but is generally in the direction of the previously found feeder, suggesting that they maintain a memory of the food source location while digging their burrow (Fig. 2). Unlike ants that start their foraging careers with short, unrewarded “learning trips” around their burrows (Fleischmann et al. 2016, 2017, 2018; Müller and Wehner 2010), *S. galenus* forage without learning forays upon their first exit from the newly built burrow. In general, one rarely sees the beetles returning home on the first and all subsequent trips without a piece of dung. The paths to the food source become straighter and faster with subsequent foraging trips, while the return paths also improve in straightness but not speed (which is likely limited by the fact that the beetles walk backwards while transporting a piece of dung) (Fig. 3). This suggests that, with repeated foraging trips, the beetles may be improving their memory of the direction to the food source and their estimate of the homeward path (by having straighter outbound paths). This gradual improvement in straightness with an increasing number of outbound trips can also be observed in ants (Fleischmann et al. 2016; Müller and Wehner 2010; Wehner et al. 2004) but, unlike ants, *S. galenus* do not make any systematic turns, loops or pirouettes or other characteristic rotations that might give them a view of their burrow as they walk away from it. By the sixth trip, the foraging paths are nearly perfectly straight, demonstrating that *S. galenus* is capable of accurately moving along a given bearing, even while moving backwards on four legs and transporting dung.

### *Scarabaeus galenus* uses path integration to return to its burrow

If *S. galenus* fail to locate their burrow at the end of their homing path, they initiate a systematic search that is characterised first by a clear deviation from the straight-line path, followed by a series of loops. The location of the turning point that indicates the start of the search pattern lies very close to the location of the real burrow (in the case where the burrow has been covered) (Fig. 4) or to the fictive burrow (in cases where the beetles had been transferred to a new location) (Fig. 7). This suggests that the beetles behave similarly whether navigating on familiar terrain or in an unfamiliar location.

If the beetles are passively displaced from the feeder location (either laterally or towards/away from the burrow), they faithfully return along a bearing that is 180° from the direction they walked to the feeder and then initiate a search at a distance that is almost exactly the same as the distance walked on the outbound journey (Fig. 5). While these results indicate that the beetles are indeed using path integration to locate their burrow (Heinze et al. 2018), they also suggest that the beetles are not using cues such as landmarks to accurately pinpoint its precise location in space. A navigating animal that uses landmarks to locate its home, will steer directly towards this location after a sideways displacement (Bühlmann et al. 2018).

Another indication that an animal may be using landmarks is the speed at which it approaches its goal. When approaching the nest, ants drastically reduce their speed when they have returned along 85% of their home vector (Bühlmann et al. 2018). This reduction in speed is hypothesised to support landmark orientation at close quarters, thereby improving the accuracy with which they can locate their nest (Bühlmann et al. 2018). Unlike ants, *S. galenus* homes at a nearly constant speed (Fig. 4e), seemingly oblivious to its approach through the familiar surroundings in the neighbourhood of its burrow.

### *Scarabaeus galenus* does not require landmarks to home

Homing beetles that were collected at their burrow and displaced back to the feeder are considered to have run off their home vector (i.e., they are in a ‘zero-vector’ state) and can no longer rely on path integration to locate their burrow. If the beetles were using the skyline panorama or landmarks to find their way home, they should nonetheless be able to locate their burrow after this displacement, as ants are able to do (e.g., Bühlmann et al. 2018). When released back at the feeder site, these zero-vector state beetles did not return home but immediately initiated their burrow search behaviour (Fig. 6), again suggesting that neither the skyline panorama, nor landmark cues play a role in the homing behaviour of *S. galenus*. Instead, they rely primarily on distance and direction cues acquired during their outbound journey, even after having performed over 6 return journeys between the same feeder and burrow. The observation that a foraging beetle, when displaced towards its open burrow on its inbound trip, will run straight past the entrance, even if they pass by within a few centimeters of it (Fig. 5b), also suggests that odor plays a very minor role in the location of the burrow, if at all. Again, this stands in contrast to the strategy employed by ants that also use olfactory cues to locate their nest entrance (Steck 2012; Steck et al. 2009, 2011).

Overall, our findings suggest that *S. galenus* does not complement its path integration with landmarks to locate its burrow. This is surprising, as the use of landmarks in addition to path integration would help to improve homing precision and provide more cues to locate the goal (Heinze et al. 2018). The only other case of an insect navigator that does not need to use landmarks is the desert ant *Cataglyphis fortis*, that forages over long distances on the vast salt pans of northern Africa (Bühlmann et al. 2011; Wehner et al. 1996). In this habitat, distinct landmarks are rare and the ants can generally navigate without them. However, it is the lack of landmarks in the natural habitat rather than the lack of ability to use them that explains this behaviour: if presented with distinct landmarks at the nest, *C. fortis* will use them to locate their nests (Wehner et al. 1996). Unlike the salt pans of northern Africa, the natural savanna habitat of *S. galenus* is rich in landmarks such as trees and grass tussocks (Fig. 1b), so an absence of landmarks in the natural habitat cannot explain why they do not play an important role in homing in *S. galenus*.

The navigation strategy of *S. galenus* represents an interesting example of homing where terrestrial cues such as the skyline panorama or landmarks may not be used. We propose that this is due to several potentially interacting features of their ecology. Firstly, *S. galenus* systematically moves backwards while homing. Ants homing backwards, dragging large items of food with them, will also behave as if uninfluenced by the learned scenery of the route (Schwarz et al. 2017). Heavily loaded ants will, however, from time to time, release their bulky forage and face in the direction of their nest (Ardin et al. 2016; Pfeffer and Wittlinger 2016b; Schwarz et al. 2017). This enables them to recognize the visual scenery and correct their path (Schwarz et al. 2017). Similar rotations towards the direction of the burrow and the landmarks surrounding it, are never observed in the pellet-loaded beetles.

The second potential reason why landmarks (or nest related odor cues) may not be particularly useful for homing in *S. galenus* is that they do not typically forage over long distances. Unlike ants that repeatedly forage over 100 m away from their nest (Müller and Wehner 1988), *S. galenus* typically forages over 0.1–2.50 m (with a median of 1.5 m) between the dung and their burrow. Perhaps over these relatively short foraging distances, the path integrator of *S. galenus* does not accumulate enough errors (Heinze et al. 2018) to require the correction afforded by using landmarks or perhaps the fitness cost of missing the burrow is less high for the beetles than for other navigators. It is interesting to note that, even in a natural ‘undisturbed’ setting, homing beetles will often, for no obvious reason, miss the entrance to their burrow. When this happens, they initiate a systematic search (MD, EB, personal observation). These observations suggest that the navigation strategy of *S. galenus* is not completely robust and may reflect the lack of precision associated with a path-integration strategy that is not backed up by landmark information.

A third reason could be that most dung beetles, including *S. galenus* as far as we know, don’t occupy long term, permanent nests. Instead they fly long distances between bouts of feeding, to new habitats, where they would have to learn a new set of landmarks for a brief, once-off, provisioning session, only to move on again once that store has been depleted.

It is interesting to note that the beetles that were either passively moved sideways with their feeder (displacement experiments), or transported under an opaque container (transfer experiments), all displayed an increase in the spread of the length of their home vector, while their direction estimate remained unchanged. This suggests that their estimate of distance may be based on idiothetic measurements, which are easily disturbed by the sudden sideways deflection from the terrain or by induced or self-induced movements during food transport. A likely candidate for this estimator is a step-based odometer, similar to the one found in ants (Wittlinger et al. 2006, 2007). An extra challenge *S. galenus* possibly face if employing such an idiothetic odometer, coupled to its legs, is that while the beetles leave their burrow in a standard six-leg tripod gate, they return on only four legs, holding the pellet of dung tightly between the last pair (Fig. 1c, e). The nature of this beetle odometer will be the focus of future work.
